# Supplementary material for: YKL40/Integrin β4 Axis Induced by the Interaction between Cancer Cells and Tumor-Associated Macrophages Is Involved in the Progression of High-Grade Serous Ovarian Carcinoma
Source: Int J Mol Sci. 2024 Oct 1;25(19):10598. doi: 10.3390/ijms251910598 (PMC11477481; doi:10.3390/ijms251910598)
Supplement: Supplementary file 1 [file ijms-25-10598-s001.zip › ijms-3219212-supplementary.pdf]

Supplementary Figure and Table

YKL40/Integrin β4 Axis Induced by the Interaction between Cancer Cells and Tumor-Associated Macrophages Is Involved in the Progression of High-Grade Serous Ovarian Carcinoma

Keitaro Yamanaka, Yu-ichiro Koma, Satoshi Urakami, Ryosuke Takahashi, Satoshi Nagamata, Masaki Omori, Rikuya Torigoe, Hiroki Yokoo, Takashi Nakanishi, Nobuaki Ishihara, Shuichi Tsukamoto, Takayuki Kodama, Mari Nishio, Manabu Shigeoka, Hiroshi Yokozaki and Yoshito Terai

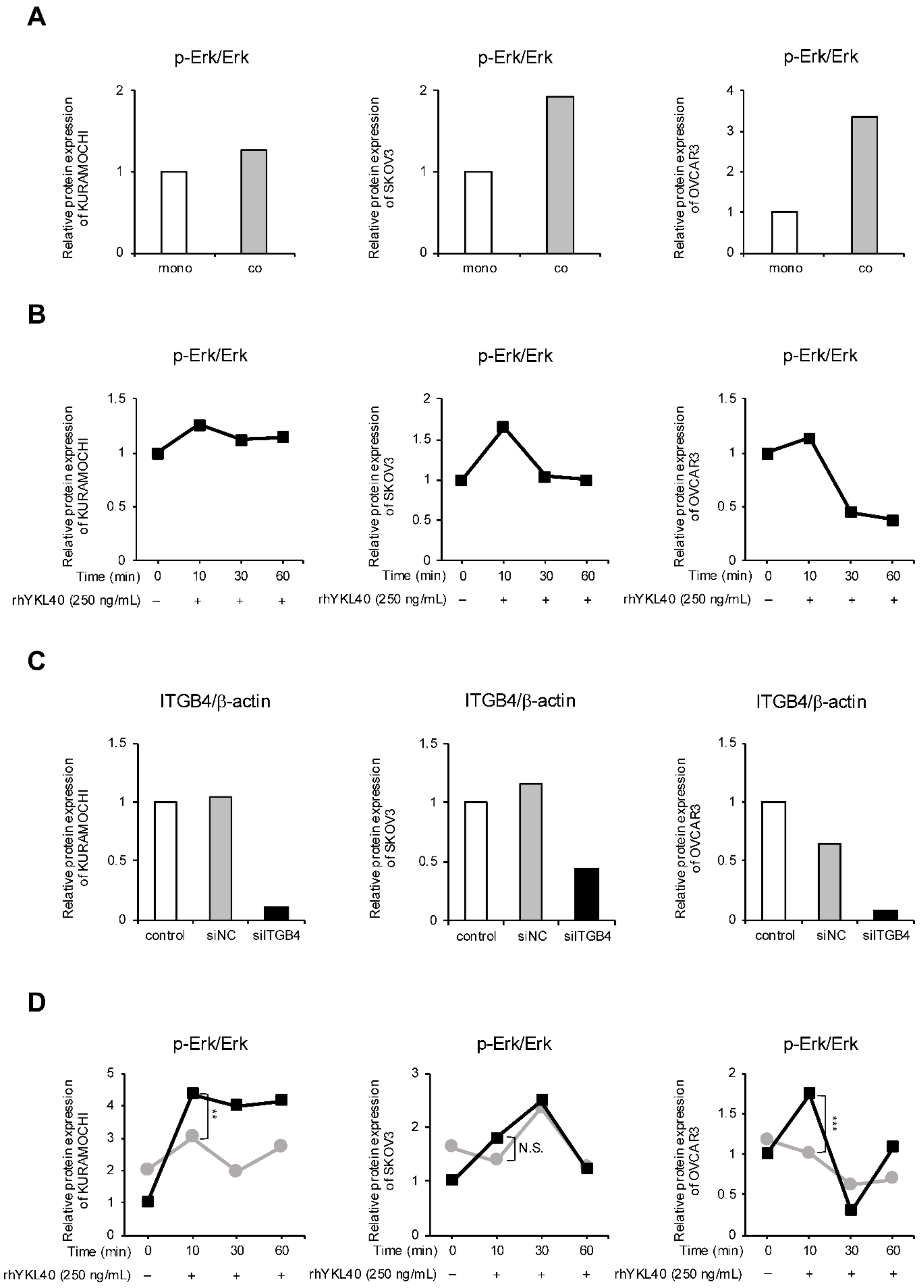

Figure S1 Quantification of bands from Western blotting shown in Figures 2C, 4A, 5B, and 5F. **A:** Expression levels of phosphorylated extracellular signal-regulated kinase (p-Erk) in epithelial ovarian cancer (EOC) cells monocultured and co-cultured with macrophages were normalized to total extracellular signal-regulated kinase (Erk) protein levels. **B:** Time-dependent expression

levels of p-Erk in EOC cells treated with recombinant human YKL40 (rhYKL40) were normalized to total Erk protein levels. **C:** Expression levels of integrin β4 (ITGB4) in EOC cells, whether non-transfected (control) or transfected with negative control siRNA (siNC) or ITGB4-targeted siRNA (siITGB4), were normalized to β-actin expression levels. **D:** Expression levels of p-Erk in EOC cells were normalized to total Erk protein levels. Plots represented by squares indicate time-dependent expression levels in EOC cells transfected with siNC, whereas plots represented by circles indicate time-dependent expression levels in EOC cells transfected with siITGB4. $^{**}P < 0.01$, $^{***}P < 0.001$. N.S., not significant.

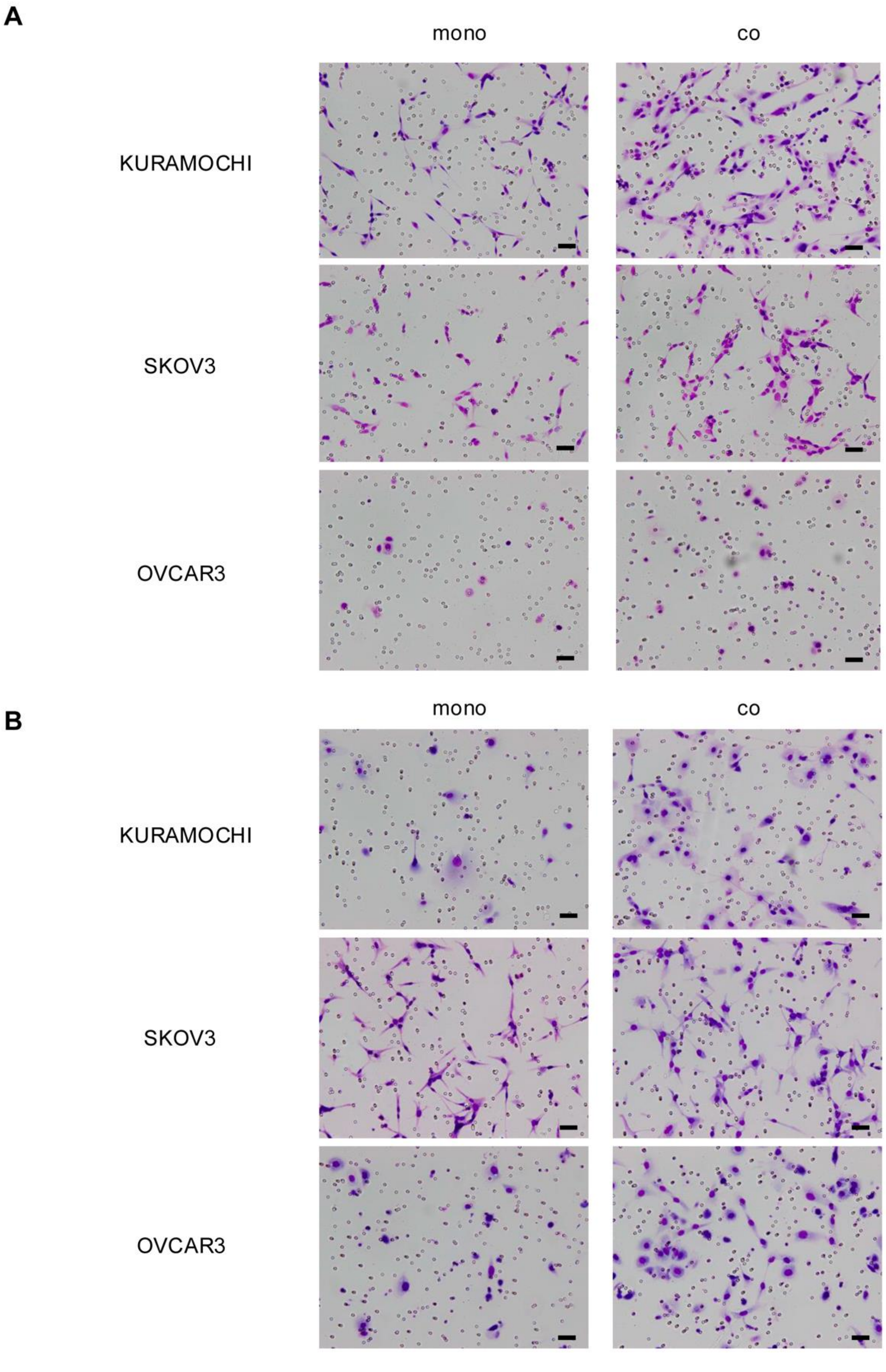
A
mono
co
KURAMOCHI
SKOV3
OVCAR3
B
mono
co
KURAMOCHI
SKOV3
OVCAR3

C

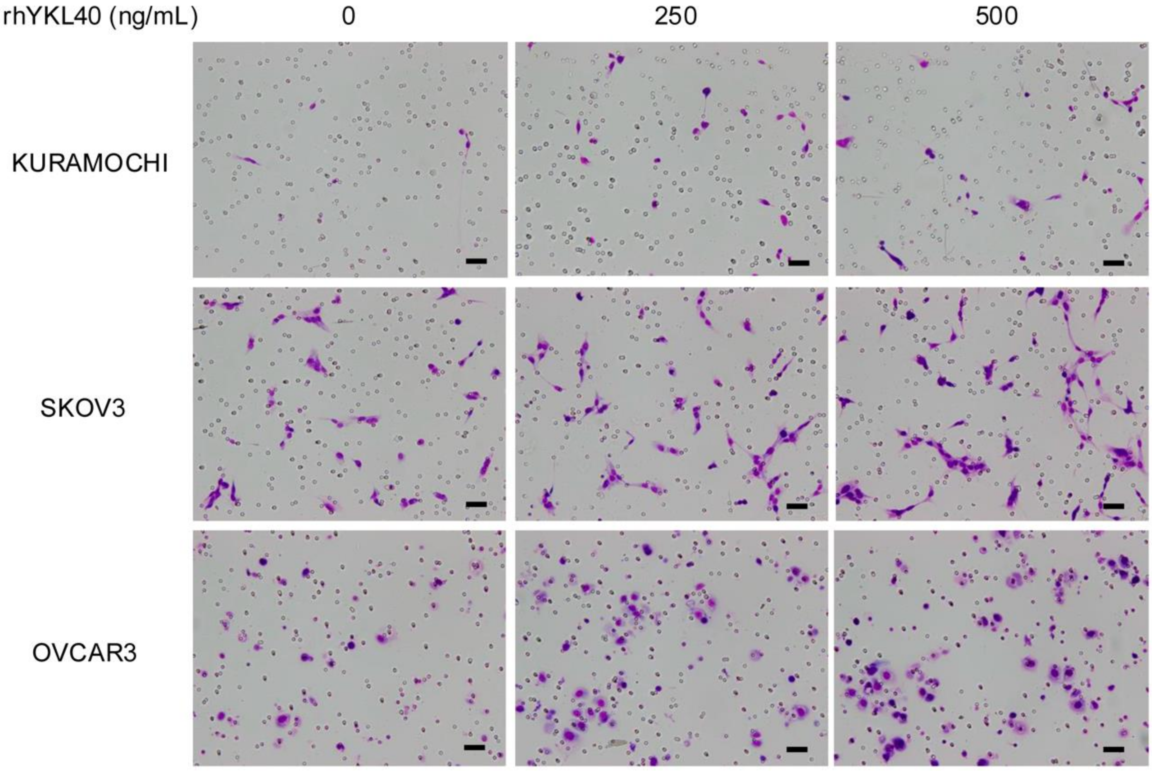

rhYKL40 (ng/mL)
0
250
500
KURAMOCHI
SKOV3
OVCAR3

D

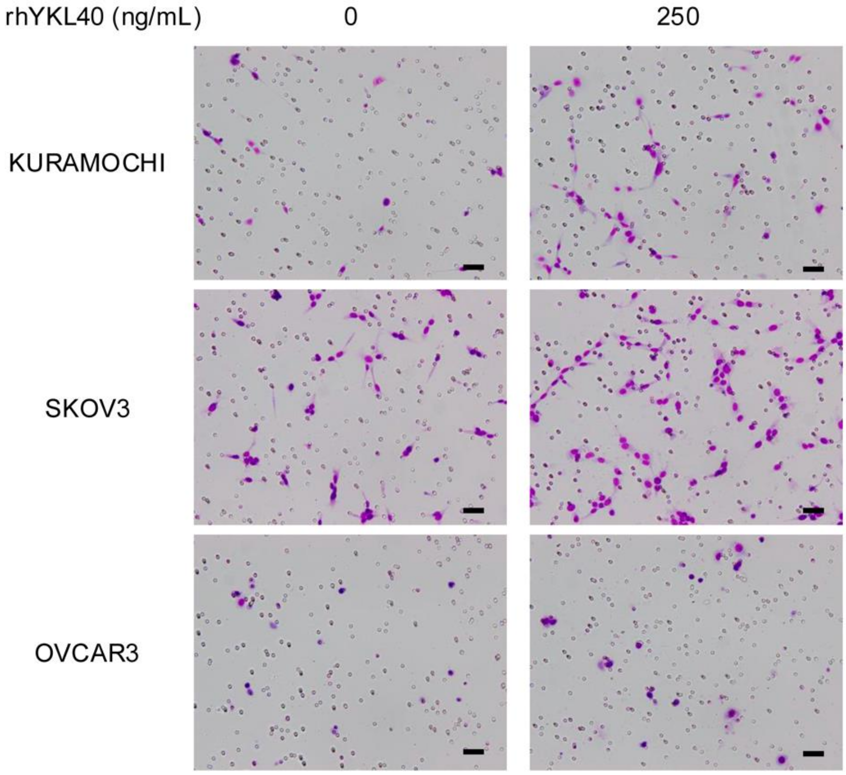

rhYKL40 (ng/mL)
0
250
KURAMOCHI
SKOV3
OVCAR3

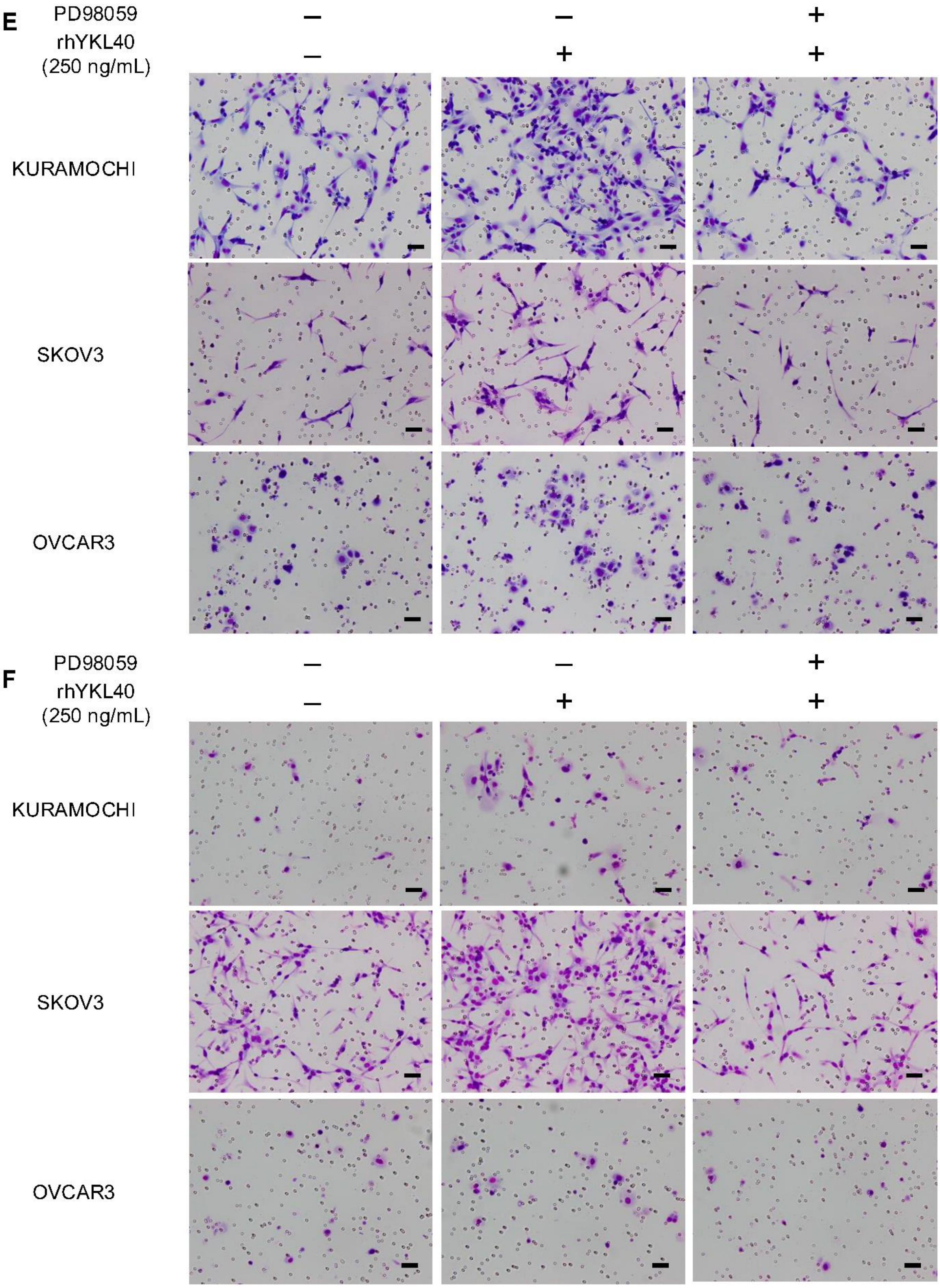
E
PD98059
rhYKL40
(250 ng/mL)
–
–
–
+
+
+
KURAMOCHI
SKOV3
OVCAR3
F
PD98059
rhYKL40
(250 ng/mL)
–
–
–
+
+
+
KURAMOCHI
SKOV3
OVCAR3

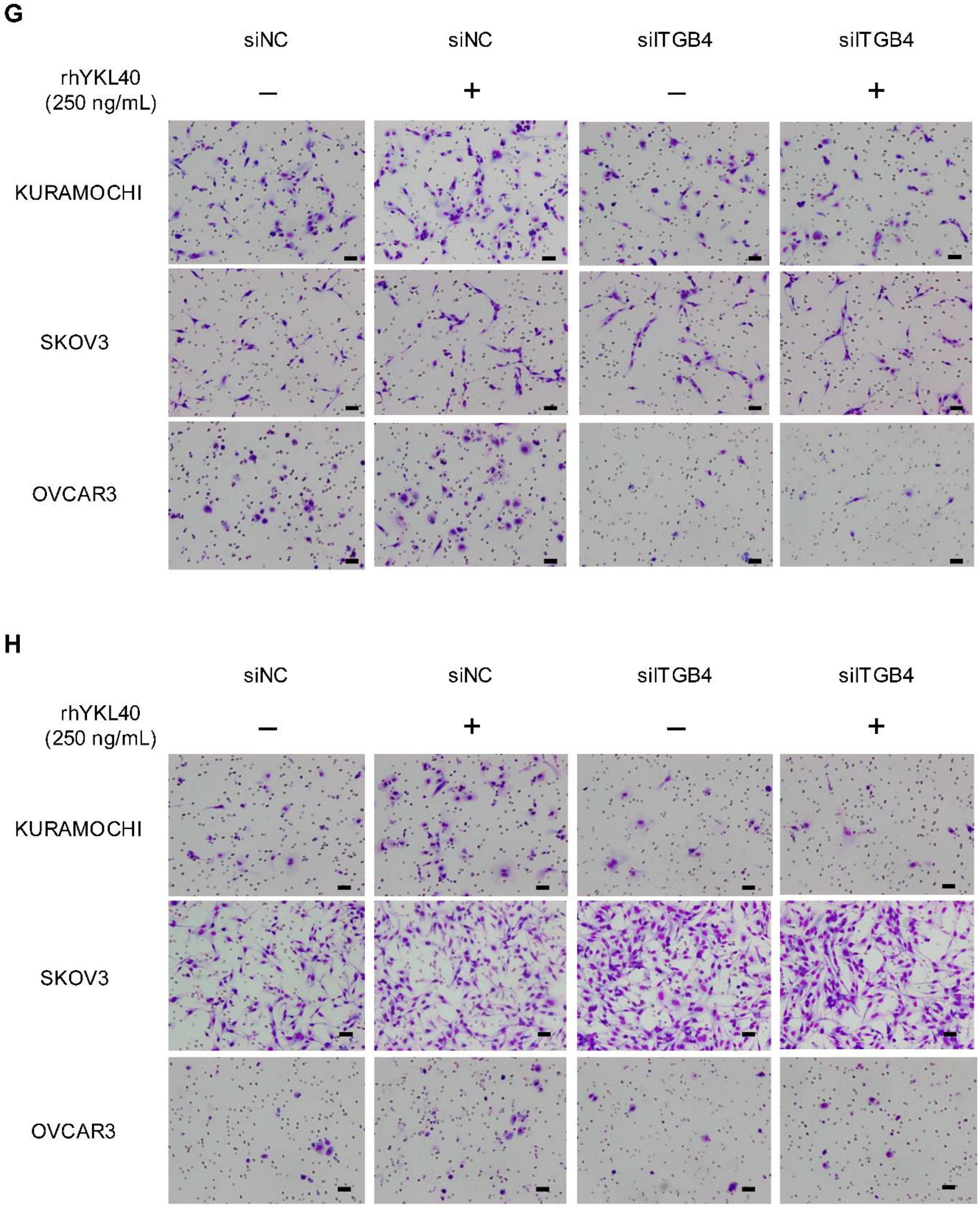

Figure S2 Representative images of transwell migration and invasion assays shown in Figures 2E (A), 2F (B), 3E (C), 3F (D), 4C (E), 4D (F), 5D (G), and 5E (H). Scale bar: 20 μm.

	1,2	3,4	5,6	7,8	9,10	11,12	13,14	15,16	17,18	19,20	21,22	23,24
A	Reference Spots	Adiponectin	Apolipoprotein	Angiogenin	Angiopoietin-1	Angiopoietin-2	BAFF	BDNF	C5/C5a	CD14	CD30	Reference Spots
B		CD40 ligand	YKL-40	Adipsin	CRP	Cripto-1	Cystatin C	Dkk-1	DPP4	EGF	EMMPRIN	
C		CXCL5	Endoglin	Fas Ligand	FGF-2	FGF-7	FGF-19	FLT3LG	G-CSF	GDF-15	GM-CSF	
D	CXCL1	GH	HGF	ICAM-1	IFN-γ	IGFBP-2	IGFBP-3	IL-1α	IL-1β	IL-1ra	IL-2	IL-3
E	IL-4	IL-5	IL-6	IL-8	IL-10	IL-11	IL-12p70	IL-13	IL-15	IL-16	IL-17A	IL-18 Bpa
F	IL-19	IL-22	IL-23	IL-24	IL-27	IL-31	IL-32	IL-33	IL-34	CXCL10	CXCL11	PSA
G	Leptin	LIF	Lipocalin-2	CCL2	CCL7	M-CSF	MIF	CXCL9	CCL3/CCL4	CCL20	CCL19	MMP9
H	MPO	OPN	PDGF-AA	PDGF-AB	PTX3	CXCL4	RAGE	CCL5	RBP-4	Relaxin-2	Resistin	SDF-1α
I	Serpin E1	SHBG	ST2	TARC	TFF3	TfR	TGF-α	THBS1	TNF-α	uPAR	VEGF	
J	Reference Spots		VDB	CD31	TIM-3	VCAM-1						Negative controls

Figure S3 Cytokine array used in this study. Coordinate of capture antibodies in Proteome Profiler Human XL Cytokine Array Kit. Green boxes show increased expression in supernatants of KURAMOCHI/macrophage co-culture compared with KURAMOCHI monoculture and macrophage monoculture.

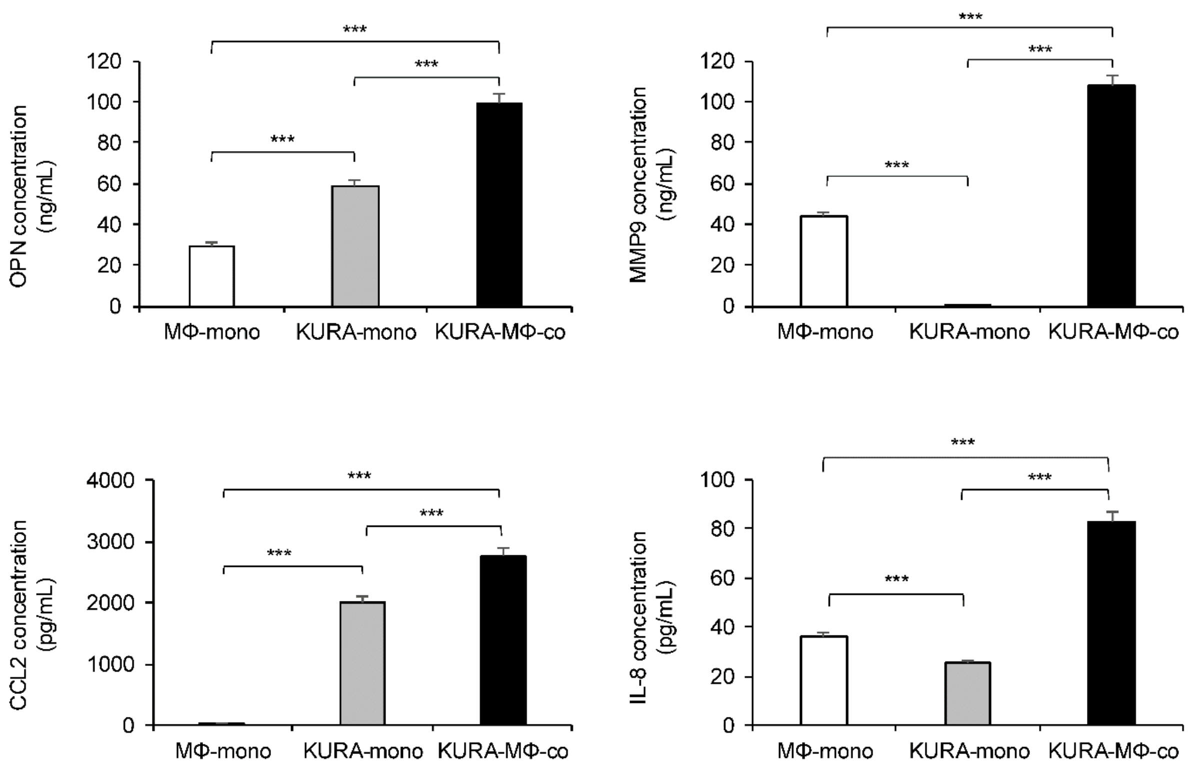

Figure S4 Increased secretion of osteopontin (OPN), interleukin (IL)-8, CCL2, and matrix metalloproteinase (MMP) 9 in KURAMOCHI/macrophage co-culture. ELISA was performed to investigate the secretion of OPN, IL-8, CCL2, and MMP9 in the supernatants of three different cultures: KURA-Mϕ-co, KURAMOCHI/macrophage co-culture; KURA-mono, KURAMOCHI cell monoculture; Mϕ, macrophage monoculture. ***$P < 0.001$.

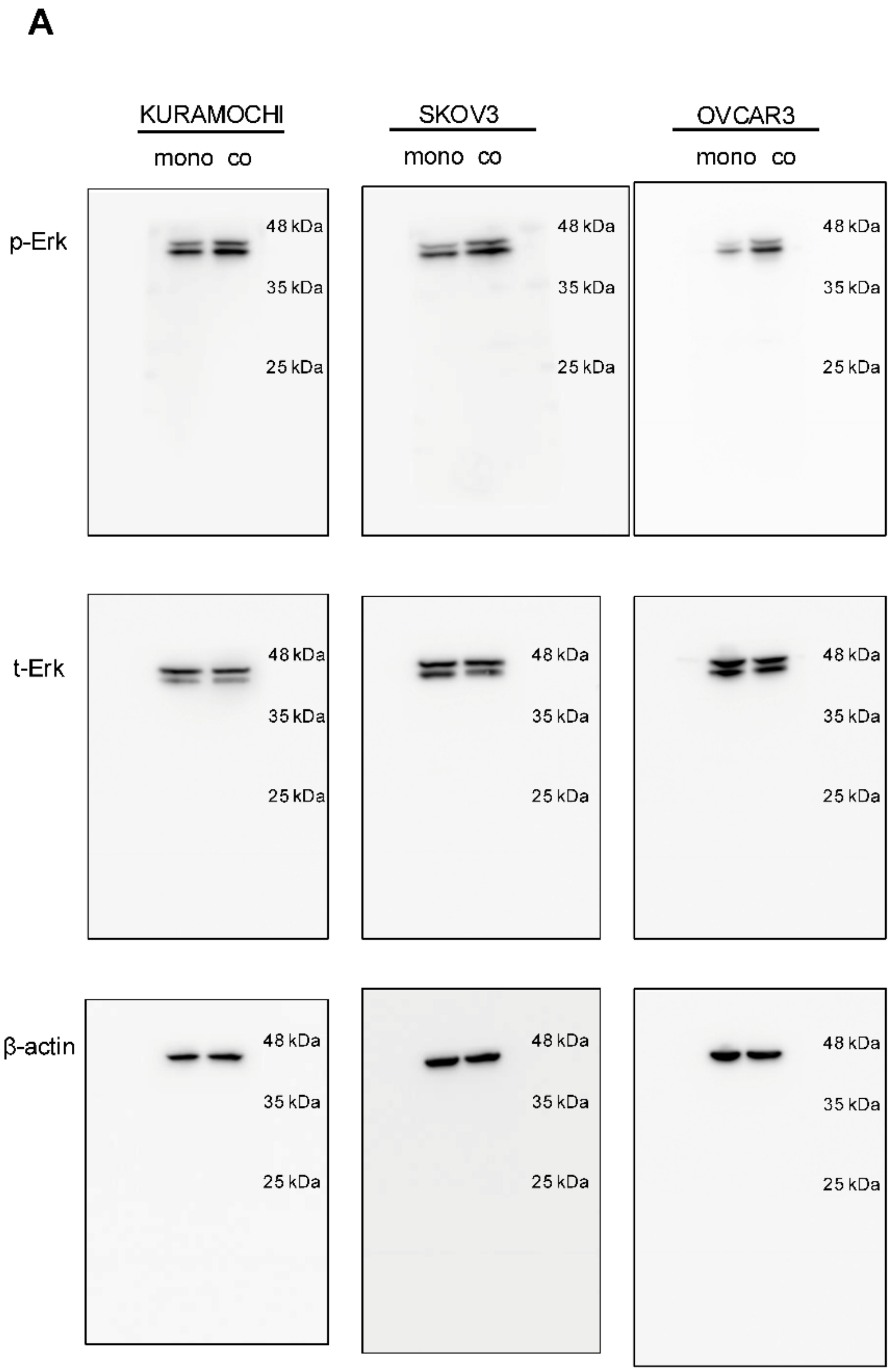
A
KURAMOCHI
SKOV3
OVCAR3
mono co
mono co
mono co
p-Erk
t-Erk
β-actin
48 kDa
35 kDa
25 kDa

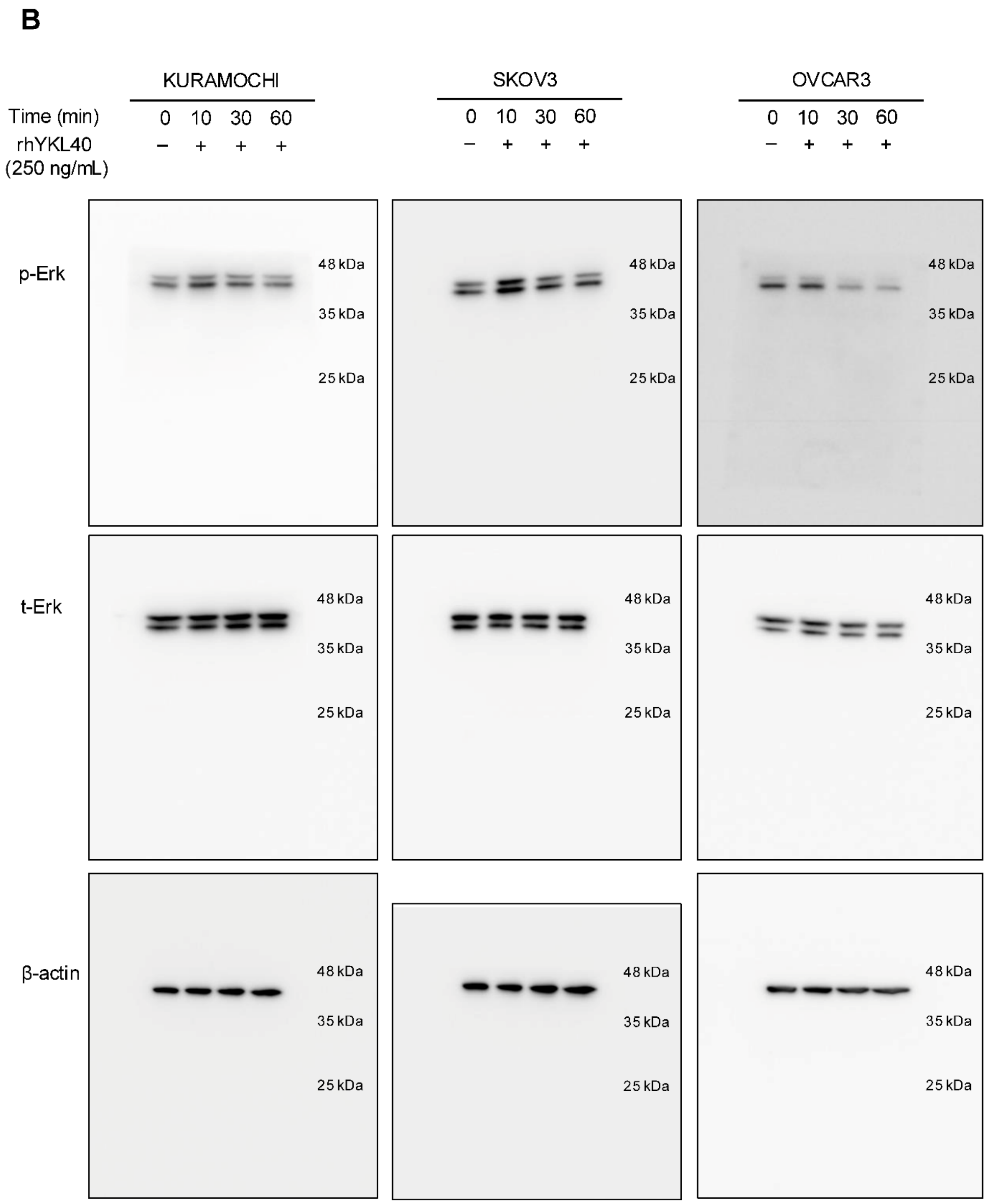
B
KURAMOCHI
SKOV3
OVCAR3
Time (min)
0 10 30 60
rhYKL40
(250 ng/mL)
− + + +
p-Erk
t-Erk
β-actin
48 kDa
35 kDa
25 kDa

C

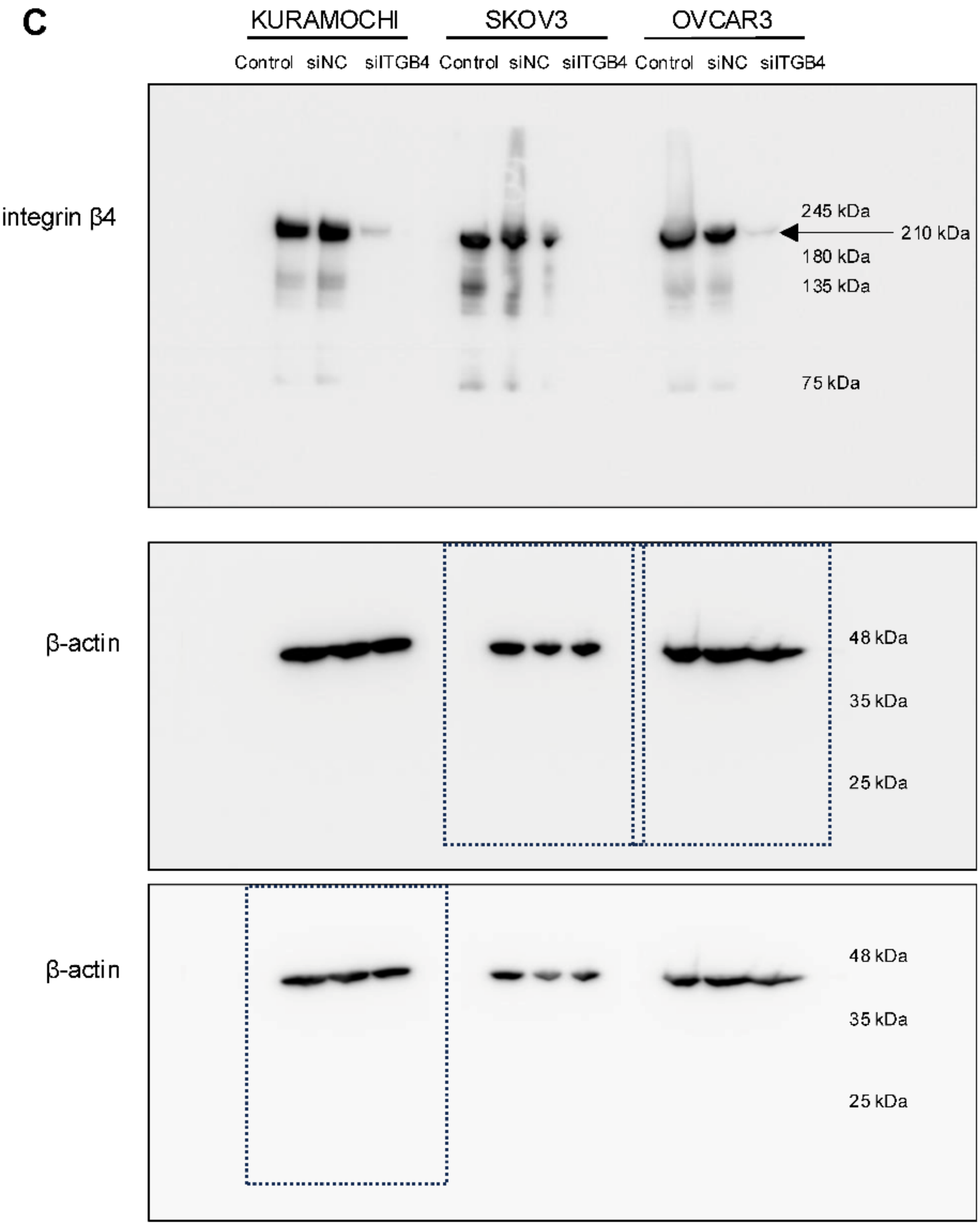

KURAMOCHI
SKOV3
OVCAR3
Control siNC siITGB4 Control siNC siITGB4 Control siNC siITGB4
integrin β4
245 kDa
210 kDa
180 kDa
135 kDa
75 kDa
β-actin
48 kDa
35 kDa
25 kDa
β-actin
48 kDa
35 kDa
25 kDa

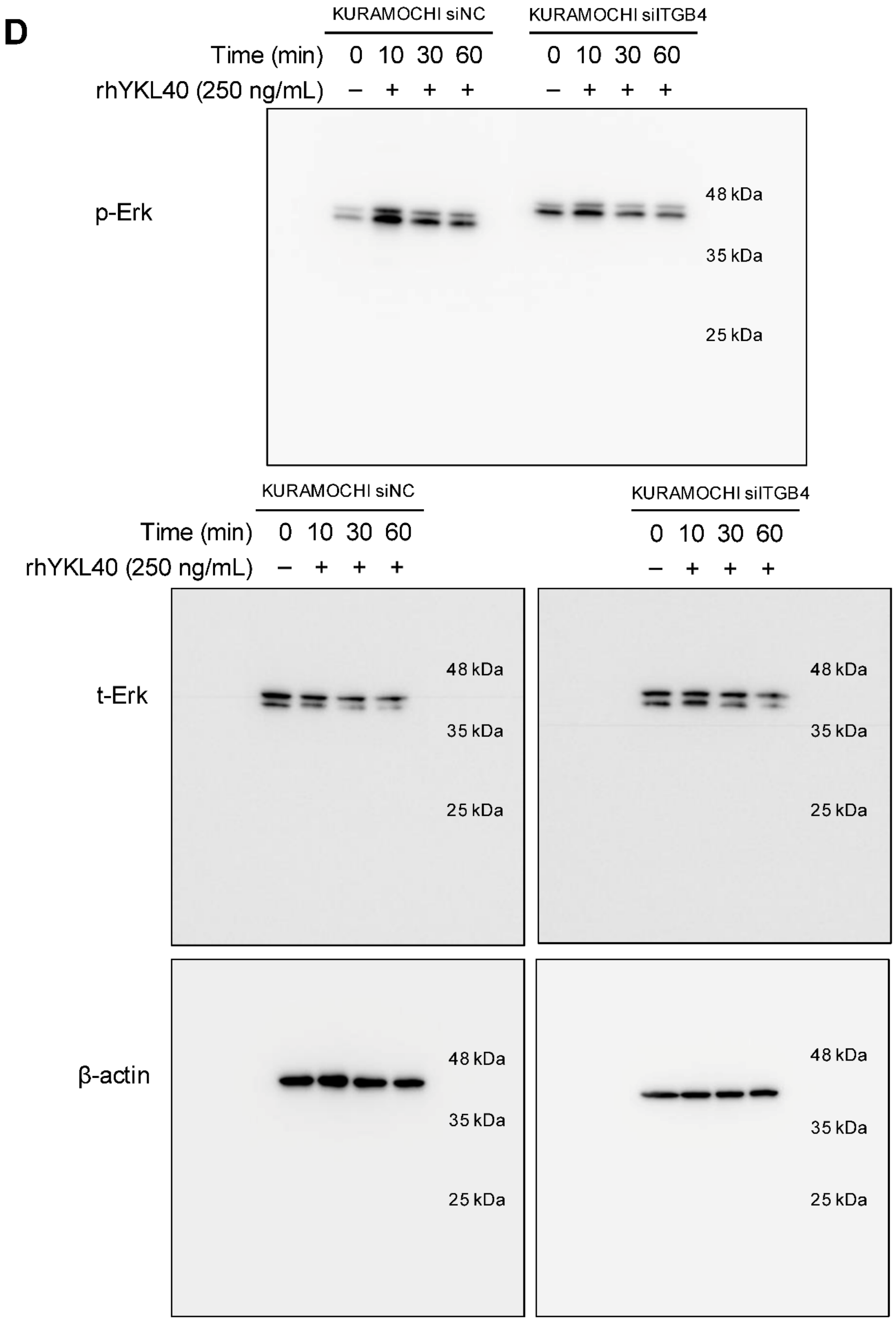

D
KURAMOCHI siNC
KURAMOCHI siITGB4
Time (min) 0 10 30 60 0 10 30 60
rhYKL40 (250 ng/mL) – + + + – + + +
p-Erk
48 kDa
35 kDa
25 kDa
KURAMOCHI siNC
KURAMOCHI siITGB4
Time (min) 0 10 30 60
rhYKL40 (250 ng/mL) – + + +
0 10 30 60
– + + +
t-Erk
48 kDa
35 kDa
25 kDa
48 kDa
35 kDa
25 kDa
β-actin
48 kDa
35 kDa
25 kDa
48 kDa
35 kDa
25 kDa

D

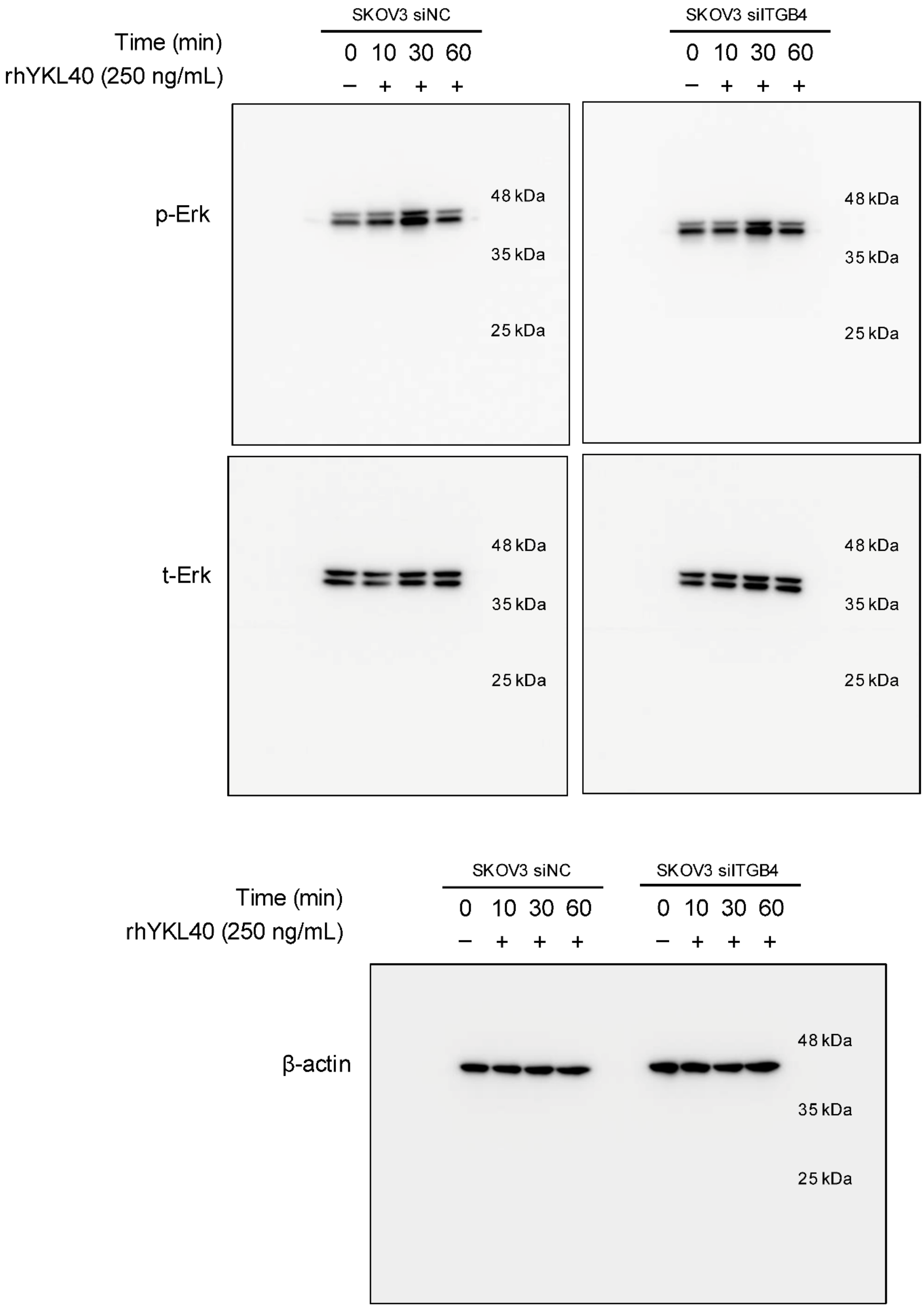

SKOV3 siNC
SKOV3 siITGB4
Time (min)
0 10 30 60
0 10 30 60
rhYKL40 (250 ng/mL)
− + + +
− + + +
p-Erk
48 kDa
35 kDa
25 kDa
48 kDa
35 kDa
25 kDa
t-Erk
48 kDa
35 kDa
25 kDa
48 kDa
35 kDa
25 kDa
SKOV3 siNC
SKOV3 siITGB4
Time (min)
0 10 30 60
0 10 30 60
rhYKL40 (250 ng/mL)
− + + +
− + + +
β-actin
48 kDa
35 kDa
25 kDa

D

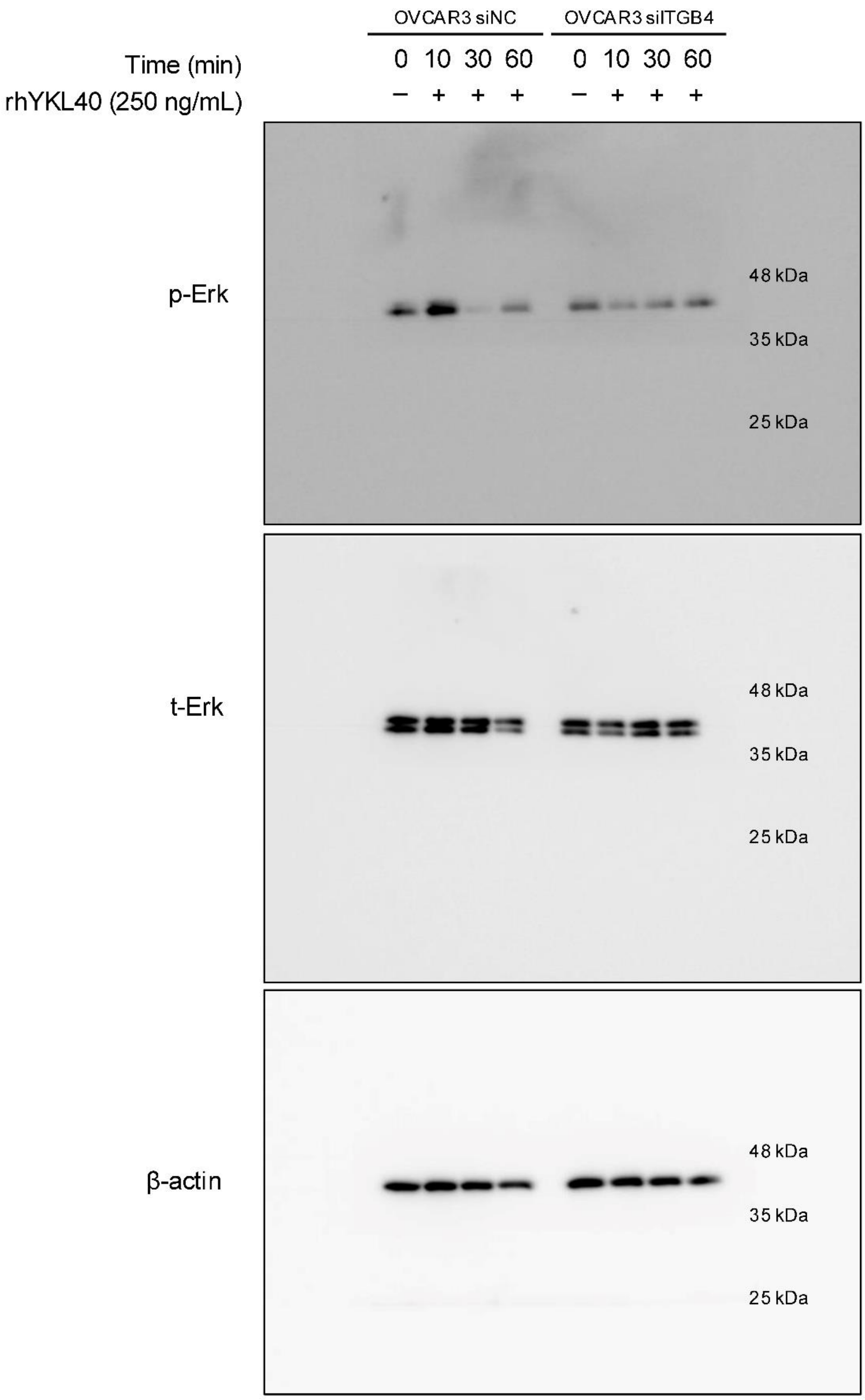

Figure S5 The original Western blotting images corresponding to Figures 2C, 4A, 5B, and 5F are provided as Figure S5A-D, respectively. The protein markers are not indicated on these original membranes.

Table S1 The sequences of the primers for qPCR.

Primers		
CD163	Forward :	5'-CGAGTTAACGCCAGTAAG-3′
	Reverse :	5′-GAACATGTCACGCCAGC-3′
CD204	Forward :	5′-CCAGGGACATGGGAATGCAA-3′
	Reverse :	5′-CCAGTGGGACCTCGATCTCC-3′
ITGB4	Forward :	5′-GCTTCACACCTATTTCCCTGTC-3′
	Reverse :	5′-GACCCAGTCCTCGTCTTCTG-3′
GAPDH	Forward :	5′-GCACCGTCAAGCCTGAGAAT-3′
	Reverse :	5′-ATGGTGGTCAAGACGCCAGT-3′
